# Supplementary material for: ADAM10 and ADAM17 promote SARS‐CoV‐2 cell entry and spike protein‐mediated lung cell fusion
Source: EMBO Rep. 2022 May 8;23(6):e54305. doi: 10.15252/embr.202154305 (PMC9171409; doi:10.15252/embr.202154305)
Supplement: Supplementary file 1 — Expanded View Figures PDF [file EMBR-23-0-s003.pdf]

Expanded View Figures

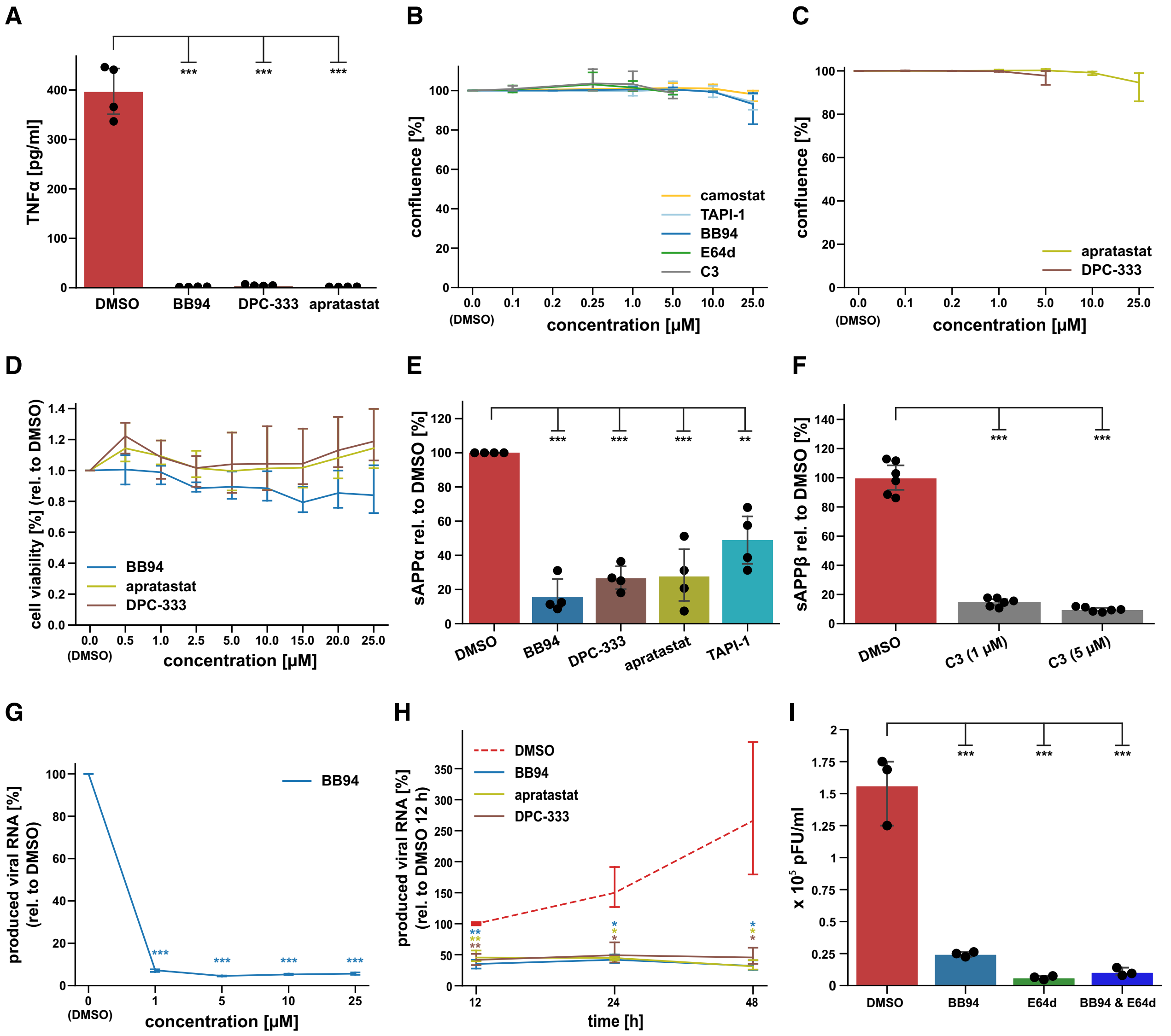

Figure EV1.

Figure EV1. Influence of inhibitors on ADAM protease activity and cell viability.

A U937 macrophages were treated with LPS (100 ng/ml) for 2 h after exposure to either DMSO or indicated inhibitor. TNFα was measured in conditioned medium by ELISA ($N = 4$). One-way ANOVA with Tukey's correction.

B Results are from the same experiment as in Fig 1D and demonstrate that inhibitors did not alter cell viability, as shown by means of cell confluence during the experiment. Data are normalized to DMSO ($N = 3$). Two-sided independent Student's t-test with Benjamini–Hochberg FDR correction was performed for every indicated concentration compared with DMSO (0 μM).

C Results are from the same experiment as in Fig 1F and demonstrate that inhibitors did not alter cell viability, as shown by means of cell confluence at the end of the experiment at 48 h. Data are normalized to DMSO ($N = 3$). Two-sided independent Student's t-test with Benjamini–Hochberg FDR correction was performed for every indicated concentration compared with DMSO (0 μM).

D In addition to Fig EV1B and C cell viability was assessed using the colorimetric MTT assay. A549-ACE2 cells were exposed to 0.5–25 μM of indicated inhibitors for 48 h. Data are normalized to DMSO ($N = 4$–6). A Two-sided independent Student's t-test with Benjamini–Hochberg FDR correction was performed for every indicated concentration compared with DMSO (0 μM).

E, F A549-ACE2 cells were treated for 48 h with 10 μM of indicated ADAM protease inhibitors or indicated concentrations of the BACE inhibitor C3. Concentration of the ADAM protease cleavage fragment of the amyloid precursor protein (sAPPα) or the ß-secretase cleavage fragment (sAPPß) was determined in the conditioned medium by ELISA and is shown relative to the concentration in the DMSO control ($N = 3$). sAPPα is known to be generated by ADAM10 and ADAM17, while sAPPß is generated by BACE1. All inhibitors significantly blocked sAPPα or sAPPß generation, demonstrating successful inhibition of ADAM or BACE proteases with the inhibitors. Two-sided independent Student's t-test with Benjamini–Hochberg FDR correction.

G Experiment was conducted and analyzed as in Fig 2A. Cells were pretreated with indicated concentrations of BB94 or DMSO for 6 h and infected with SARS-CoV-2 (MUC-IMB-1). RNA was isolated 24 h postinfection. Data are normalized to DMSO ($N = 3$). Two-sided independent Student's t-test with Benjamini–Hochberg FDR correction was performed for every indicated concentration compared with DMSO (0 μM).

H Experiment was conducted as described in Fig 2A. Cells were pretreated with BB94 (10 μM), apratastat (10 μM), DPC-333 (5 μM), or DMSO for 6 h. RNA was isolated at indicated time points after infection. Inhibitor treatment reduced infection of A549-ACE2 cells up to 48 h postinfection, at which time the experiment was terminated due to virus-induced cytopathic effects. Data are normalized to DMSO 12 hpi ($N = 3$). Two-sided independent Student's t-test with Benjamini–Hochberg FDR correction was performed compared with DMSO of the respective time point.

I A549-ACE2 cells pretreated for 6 h with either DMSO or respective inhibitors (BB94 = 10 μM, E64d = 5 μM, or both) and then infected with SARS-CoV-2 at MOI 1. 24 h postinfection, produced infectious progeny was tittered on VeroE6 cells. Data are expressed as plaque-forming units (pFU) per ml of virus containing supernatant ($N = 3$). One-way ANOVA with Tukey's correction.

Data information: All data are represented as mean ± 95% CI of at least three biological replicates. $^{**}P < 0.01$, $^{***}P < 0.001$.
Source data are available online for this figure.

 © 2022 The Authors

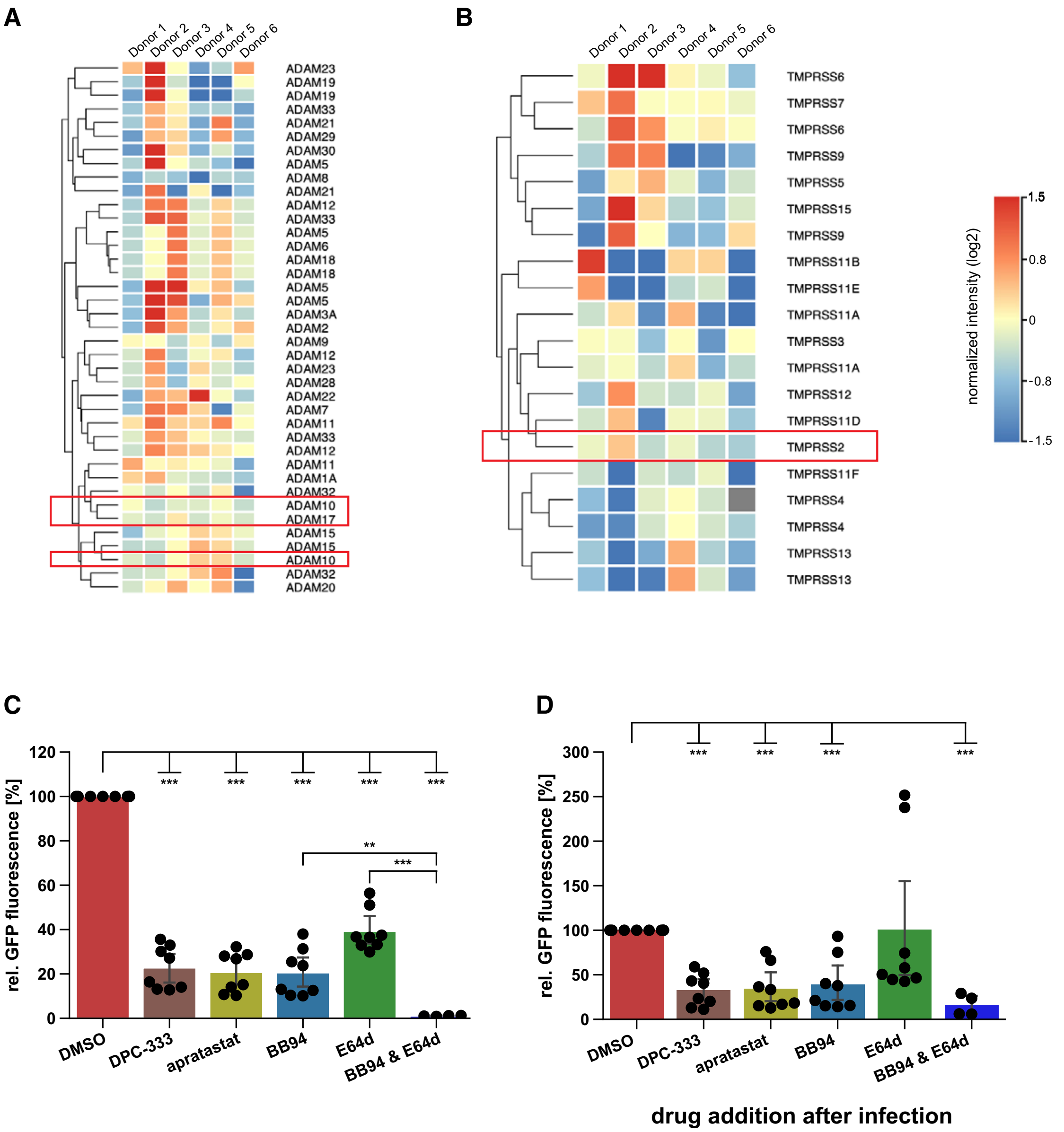

Figure EV2. Expression of ADAM and TMPRSS family members in NHBE cells.

A, B Analysis of expression levels of different ADAM and TMPRSS family members in NHBE cells (Jakwerth *et al*, 2022). Heat maps of gene expression levels for the (A) ADAM family and (B) TMPRSS family in NHBE cells from six genetically independent donors are displayed. Expression levels are given in $\log_2$ scale of normalized intensity values (see color gradient) ranging from low (blue) to high expression (red). Hierarchical clustering on entities was performed using Manhattan similarity measure. Duplicate gene names indicate two or more transcripts/isoforms of the same gene.

C, D A549-ACE2 cells were infected with GFP-expressing SARS-CoV-2. Infection of cells was monitored microscopically as green fluorescence through live-cell imaging at 48 h postinfection (hpi) and is shown as the mean of the GFP-positive area relative to the whole area covered by cells in the same well. In (C) A549-ACE2 cells were pretreated with the indicated drugs for 6 h before infection or were treated in (D) with the indicated drugs 4 h after infection. Data are normalized to DMSO (N = 4–8). Two-sided independent Student's t-test with Benjamini–Hochberg FDR correction. All data are represented as mean ± 95% CI from at least four independent experiments. $^{**}P < 0.01$, $^{***}P < 0.001$.

Source data are available online for this figure.

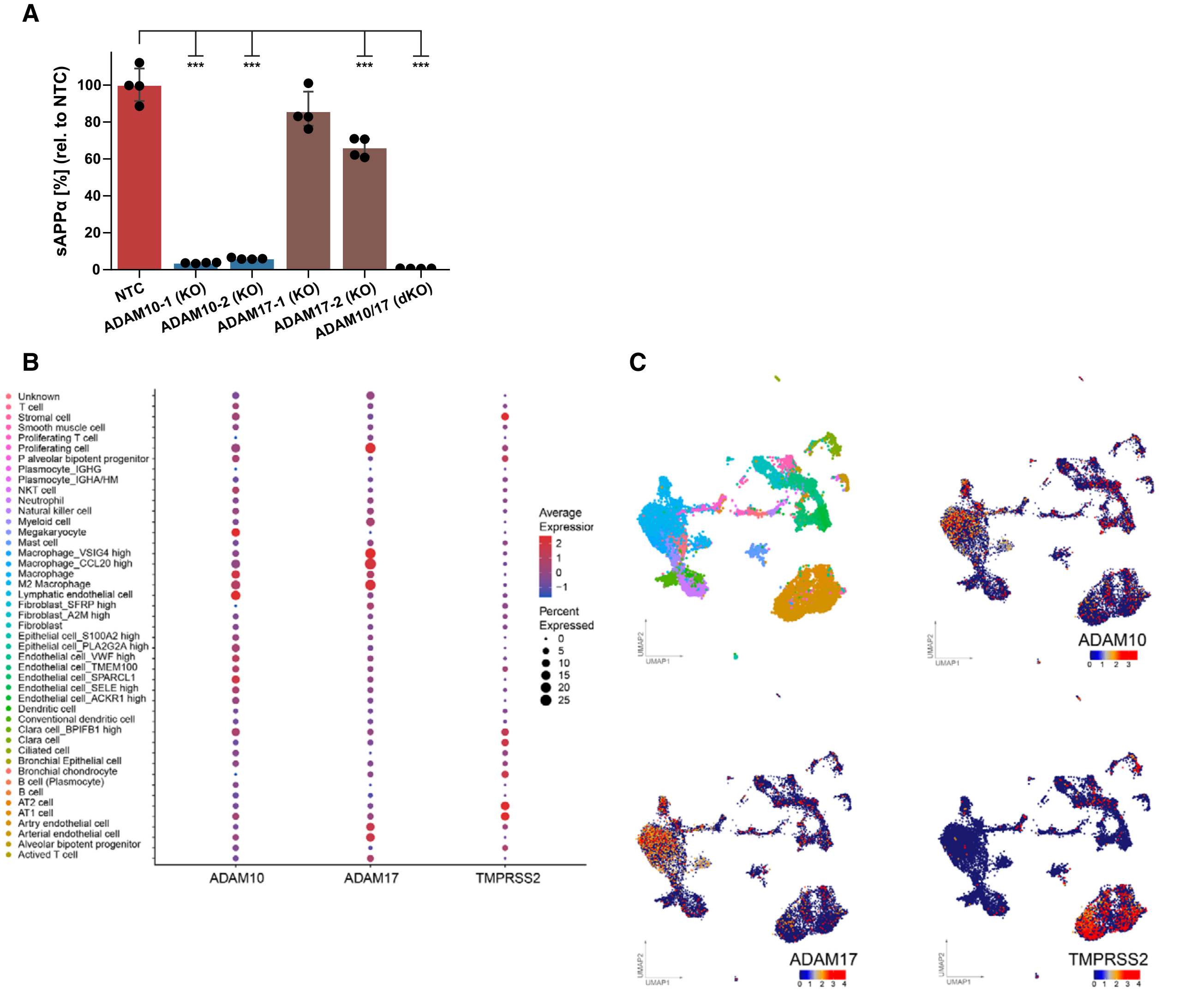

Figure EV3. Expression of ADAM10, ADAM17, and TMPRSS2 in healthy lung.

A Concentration of sAPPα was determined in the conditioned medium of A549-ACE2 NTC or KO cells by ELISA and is shown relative to the concentration of the control cells (NTC; $N = 4$). The results demonstrate that ADAM protease activity was functionally depleted in the indicated KO cells because sAPPα, which is mainly shed by ADAM10 and to a lesser extent by ADAM17, was reduced in the supernatants of the single and the double KO cells. Two-sided independent Student's *t*-test with Benjamini–Hochberg FDR correction. Data are represented as mean ± 95% CI of four independent experiments. $^{***}P < 0.001$.

B, C Dot plot (B) of the expression of ADAM10, ADAM17, and TMPRSS2 in lung cell subtypes from healthy individuals. Expression levels are color-coded, the percentage of cells expressing the respective genes is size-coded. UMAPs (C) showing the lung cell subtypes and expression levels of ADAM10, ADAM17, and TMPRSS2. Each dot represents a single cell (cell number $N = 17{,}438$). The cell cluster identity noted on the color key is based on annotation provided by Han *et al*.

Source data are available online for this figure.

 © 2022 The Authors

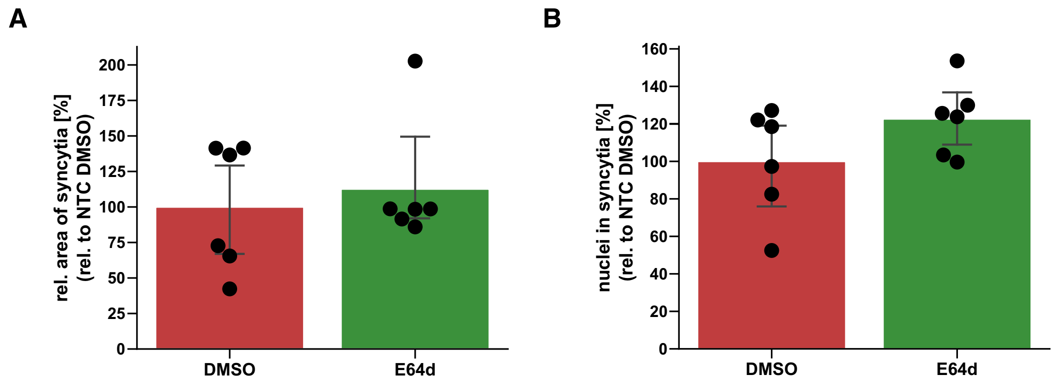

Figure EV4. E64d does not prevent syncytia formation.

A, B Quantification of syncytia. The experiment was conducted and analyzed as described in Fig 4. The area of the fused cells (GFP-positive) was quantified and normalized to the Hoechst signal of the entire image to account for differences in cell density (A). The nuclei within syncytia were determined by calculating the ratio between the Hoechst signal within syncytia and the total Hoechst signal in the entire image (B). The data are normalized to NTC DMSO ($N = 6$). Two-sided independent Student's *t*-test with Benjamini–Hochberg FDR correction. Data are represented as mean ± 95% CI of three independent experiments (six images per condition were analyzed).

Source data are available online for this figure.

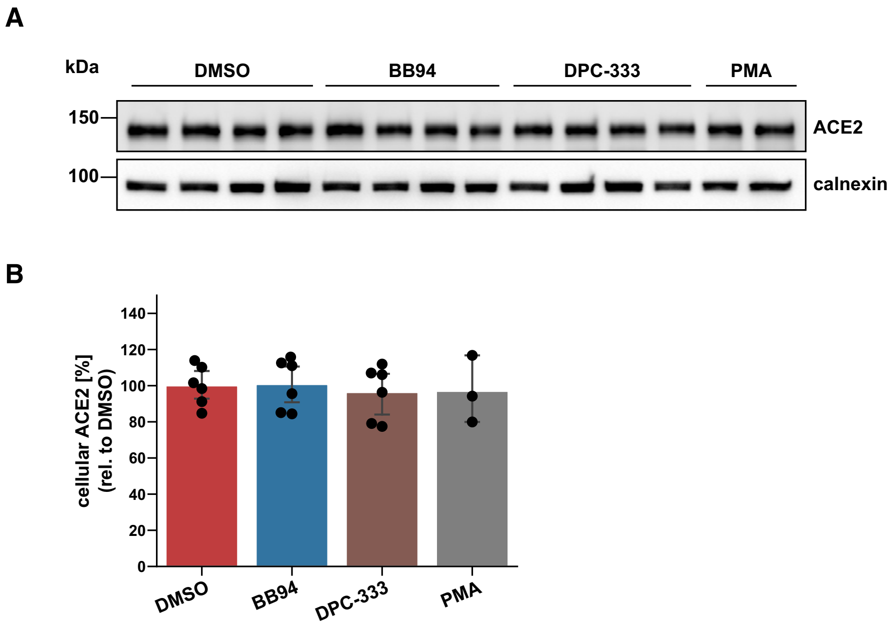

Figure EV5. Cellular ACE2 levels remain unaffected upon chronic treatment with ADAM protease inhibitors.

A, B Western Blot analysis of cellular ACE2. Results are from the same experiment as in Fig 5C. A549-ACE2 cells were treated for 48 h with BB94 (10 μM), DPC-333 (5 μM), or DMSO. PMA (25 ng/ml) was added for 3 h before harvest to stimulate ACE2 shedding. The results demonstrate that cellular ACE2 levels remained unaffected over the course of the experiment. Data are normalized to loading control calnexin and DMSO ($N = 3$–6). Two-sided independent Student's *t*-test with Benjamini–Hochberg FDR correction. Data are represented as mean ± 95% CI of at least three biological replicates.

Source data are available online for this figure.

© 2022 The Authors
